# Myxoinflammatory Fibroblastic Sarcoma: A Radiographical, Pathological, and Immunohistochemical Report of Rare Malignancy

**DOI:** 10.1155/2015/620923

**Published:** 2015-05-18

**Authors:** Michitaka Kato, Takuji Tanaka, Takatoshi Ohno

**Affiliations:** ^1^Department of Orthopedic Surgery, Gifu Municipal Hospital, 7-1 Kashima-cho, Gifu 500-8513, Japan; ^2^Department of Diagnostic Pathology, Gifu Municipal Hospital, 7-1 Kashima-cho, Gifu 500-8513, Japan; ^3^Department of Orthopedic Surgery, Gifu University School of Medicine, 1-1 Yanagido, Gifu 501-1194, Japan

## Abstract

Myxoinflammatory fibroblastic sarcoma (MIFS) is a rare, painless, and intermediate (rarely metastasizing) fibroblastic tumor, which commonly occurs in the extremities, with an equal sex predilection. This sarcoma is composed of a mixed inflammatory infiltrate along with spindled, epithelioid, and bizarre tumor cells in a background of hyaline and myxoid areas. In spite of such a distinctive morphology, the tumor can be a diagnostic challenge, simulating inflammatory conditions as well as neoplastic nature. For accurate diagnosis, the tumor requires extensive clinical, radiological, and pathological investigations. We present a case of MIFS in a 19-year-old female who presented with a mass in the left ankle. After appropriate excision and postoperative radiation therapy, she is free of disease, including recurrence and metastasis, at 12 years postoperatively.

## 1. Introduction

Myxoinflammatory fibroblastic sarcoma (MIFS) is a rare intermediate (rarely metastasizing) fibroblastic tumor affecting males and females equally in the 40th and 50th decades of life. Described for the first time in three independent reports [1–3] in 1998, this tumor mostly affects the hands and feet. Histopathologically, MIFS consists of modified fibroblasts and has a propensity for local recurrence [4] and distant metastasis [5]. Although initially described in the acral sites, it has now been increasingly recognized in the proximal soft tissues of the limbs [6, 7]. This tumor has broad differential diagnosis [6, 7]. It can often be mistaken for various reactive fibroinflammatory processes and tumors with higher metastatic potential [1, 8]. Differential diagnoses thus include myxoid malignant fibrous histiocytoma, ganglion cysts, tenosynovitis, and spindle cell tumor. The prominent inflammation and fibrosis seen histologically in MIFS simulate a reactive process. The presence of myxoid foci and scattered bizarre cells which are occasionally multivacuolated may cause confusion with malignant fibrous histiocytoma and liposarcoma. A correct diagnosis is thus important to avoid unnecessary procedures and for proper clinical management [1].

We recently have encountered a case of MIFS in the left ankle of a 19-year-old female. Her swollen left ankle was initially diagnosed as “tenosynovitis” by an open biopsy, although a few of atypical and multivacuolated cells were scattered in the lesion. Based on histopathology of an additional intralesional excision suggesting a low-grade sarcoma, she received reexcision and thereafter radiation therapy. Twelve years after the surgery, local recurrence was not found. Our case will provide useful information regarding accurate diagnosis and management of MIFS to orthopedic surgeons, pathologists, and radiologists, because over 10-year follow-up cases have not been reported.

## 2. Case Report

A 19-year-old Japanese female presented with painless swelling with local heat in the lateral aspect of her left ankle. She first noticed the swelling 3 months before this medical examination. She had no history of trauma on her feet and ankles. There was no skin color change overlying the mass. Her ankle Range of Motion (ROM) was unrestricted and full.

**Figure 1:**
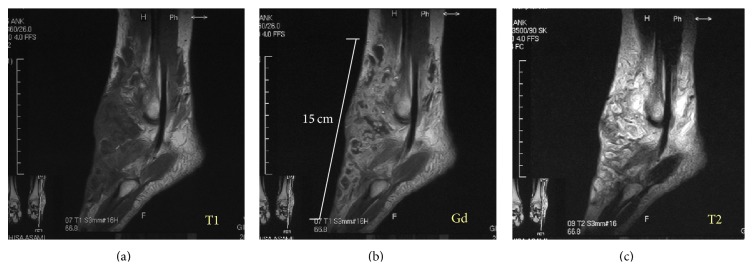
MRI showing an ill-defined heterogeneous soft tissue mass. T1 weighted image (a) shows the lobular low signal area in ill-defined border from iso- to low signal lesion, where it is enhanced by gadolinium (b) on the anterolateral part of the ankle. T2 weighted image (c) shows ill-defined delineated mixed high signal and low signal lesion.

**Figure 2:**
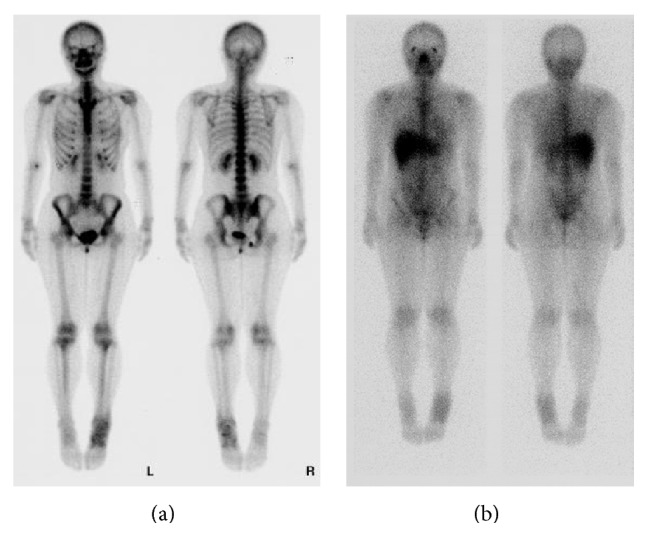
Bone (a) and Ga (b) scintigraphy show an increased uptake in the left ankle.

Plain radiographs of the left ankle and foot showed unremarkable findings except for osteolytic lesion (about 5 mm in diameter) of the navicular bone. The results of routine laboratory tests including C-reactive protein level (0.2 mg/dL) and white blood cell count (4600/*μ*L) were within normal limits. Computed tomography (CT) scans and magnetic resonance imaging (MRI) showed an ill-defined heterogeneous soft tissue mass (the main axis, 17 cm) in the subcutaneous tissue from the anterior to the lateral of the left ankle. There was a nodular or lobular mass showing T1WI low signal (Figure 1(a)), T2WI high signal (Figure 1(b)), and gadolinium- (Gd-) enhanced T1WI heterogeneous enhancement (Figure 1(c)). Bone scintigraphy (Figure 2(a)) and Ga scintigraphy (Figure 2(b)) showed increased uptake in the left ankle. The mass was slightly increased after her first visit.

**Figure 3:**
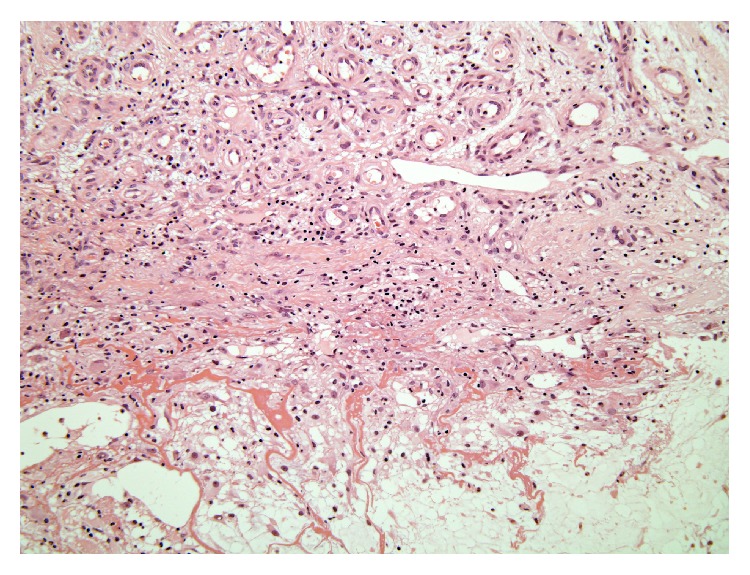
The 1st biopsied abnormal tissue shows inflammatory and edematous thickened tendon sheath with many inflammatory cells. Pathological report was “tenosynovitis.” H&E stain, ×100.

**Figure 4:**
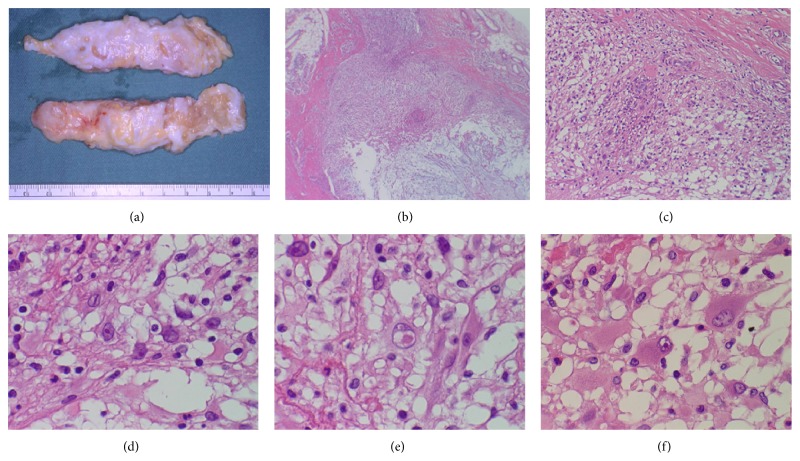
Macroscopic view of the removed tumor (a). Note the multinodular or lobular appearance of the surface. Microscopically, multinodular areas (b) were composed of alternating fibrous and myxoid areas of a proliferation of spindle or oval cells having vesicular nuclei (c) and ample eosinophilic cytoplasm (d) together with occasional larger atypical vacuolated cells, bizarre ganglion-like, Reed-Sternberg-like cells, or Hodgkin-like cells possessing prominent eosinophilic nucleoli ((e), (f)). ((b)–(f)) H&E stain; magnification, (b) ×20, (c) ×50, and ((d)–(f)) ×200.

For pathological diagnosis, an incisional biopsy (Figure 3) was performed. Histological examination of 5 mm cubic abnormal tissue biopsied showed inflammatory and edematous thickened tendon sheath with infiltration of inflammatory cells (lymphocytes and plasma cells). Based on the diagnosis “tenosynovitis (Figure 3),” the patient received antibiotics for 4 weeks, but the treatment was ineffective. Therefore, reexcision of the residual mass measuring 9.5 × 5 cm (Figure 4(a)) was performed with intralesional margin on October 2003.

## 3. Histopathology of Reresected Tumor

**Figure 5:**
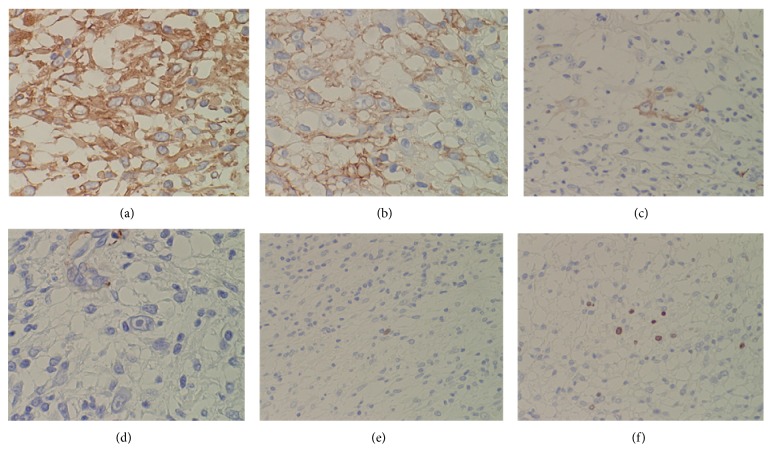
Immunohistochemistry against various antibodies. Sarcoma cells are positive for vimentin (a) and CD34 (b). A few tumor cells are positive for CD68 (c) and S-100 (d). Positive rates of nuclear p53 (e) and MIB-1 (f) were 1% and 7.5%, respectively. Immunohistochemistry, ((a)–(d)) ×200 and ((e), ( f)) ×100.

The multinodular or lobular lesion was composed of alternating fibrous and myxoid areas of proliferation of spindle or oval cells having vesicular nuclei and ample eosinophilic cytoplasm (Figures 4(b) and 4(c)) together with occasional larger atypical vacuolated cells or bizarre ganglion-like cells possessing prominent eosinophilic nucleoli (Figures 4(d), 4(e), and 4(f)), resembling Reed-Sternberg cells or Hodgkin's cells, associated with deposition of an eosinophilic fibrinous or hyaline material and a moderate chronic inflammatory infiltrate partially forming lymphoid follicles. Mitotic figures are few. Immunohistochemically, tumor cells were positive for vimentin (Figure 5(a)), CD34 (Figure 5(b)), and AE1/AE3. Some of the spindle or oval tumor cells were positive for CD68 (Figure 5(c)) and S-100 (Figure 5(d)), demonstrating a possible histiocytic component. On the other hand, tumor cells were negative for *α*-smooth muscle actin (SMA), epithelial membrane antigen (EMA), and neuron-specific enolase (NES). Percentages of p53- (Figure 5(e)) and MIB-1- (Figure 5(f)) positive cells were 1% and 7.5%, respectively.

**Figure 6:**
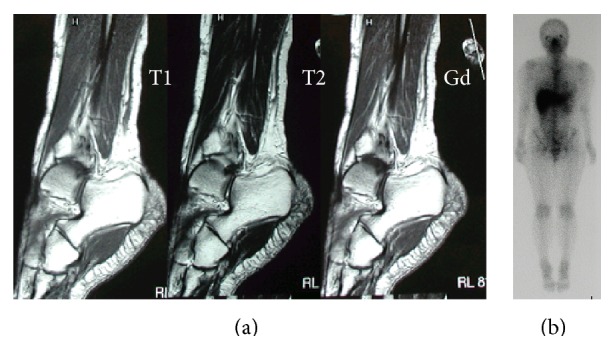
MRI (a) and bone scintigraphy (b) at 14 months after the surgery show no abnormal findings showing recurrence and local invasion of the sarcoma.

To determine whether sarcoma remained, MRI with Gd enhancement of the left ankle was performed. The MRI detected abnormal subcutaneous tissues at distal and proximal end of previous incisional area. Additional marginal margin resection of the sarcoma was performed. The skin nearby the sarcoma was preserved. In spite of the pathological report of MIFS, a wide excision was not performed because of a low-grade sarcoma. Instead, we planned radiation therapy (50 Grays). At 41.4 Grays, skin flare was observed and radiation therapy was stopped. The patient was followed up by MRI (Figure 6(a)) and bone scintigraphy (Figure 6(b)) for a period of 14 months and had no complaints and tumor recurrence.

At the time of this writing (May 2015), the patient continues to remain disease-free after twelve years of surgical excision with no evidence of recurrence or metastasis.

## 4. Discussion

A new entity and an intermediate (rarely metastasizing) fibroblastic proliferation [7, 9], MIFS, mainly occur in fingers and hands or, rarely, in feet and ankles of middle-aged patients and near equally in both genders [1, 3]. Approximately 10% of the cases were reported to be under 12 or over 75 years of age [10]. The tumor has a prominent inflammatory component and variable numbers of epithelioid tumor cells with abundant cytoplasm, large nuclei, and inclusion-like nucleoli but lacks widespread nuclear atypia and atypical mitoses. Most of the patients are asymptomatic. The tumor has a predilection for the dorsal aspect of the hands and feet, where it typically forms an ill-defined, raised mass that is much wider than deep. It may clinically resemble inflammation or infection more than a discrete tumor [6, 11]. Indeed, the initial pathological report in our case was “tenosynovitis.” Thus, there are many differential diagnoses, and it can often be mistaken for several different inflammatory and neoplastic processes, which may require different treatment [6, 7].

Grossly, the tumor typically measures 1 to 5 cm, with the median size around 3 cm and mucoid character, and is usually poorly circumscribed [6, 7]. Macroscopic features of our case resembled those of reported cases, but the size was bigger than those of previous cases [1–3]. Microscopic features contain three major characteristics: (a) multinodular architecture, alternating densely cellular and myxoid hypocellular regions; (b) mixed inflammatory infiltrate; and (c) bizarre giant and lipoblast-like cells [12]. Histopathology of the final removed tumor tissue in our case fulfilled the criteria. Although the immunohistochemical findings are nonspecific, immunohistochemistry is used for accurate diagnosis of the tumor [13]. A large numbers of histiocytes, but not large epithelioid cells, are positive for CD68 and CD163. In 10~15% of cases, large atypical cells are focally positive for keratin and EMA. The tumor cells are negative for desmin, *α*-SMA, CD34, and S-100 protein. PCR-based assays for cytomegalovirus and Epstein-Barr-virus (EBV) are negative [3]. Immunohistochemical findings in our case were almost similar to those reported previously [6, 7].

The pathogenesis of MIFS is unknown. As noted in our case, no infectious agents, including cytomegalovirus (CMV) and EBV, have been identified [3, 6, 14]. In cytogenetic studies, an unbalanced translocation t(1;10)(p22;q24) and 3p amplification involving the VGLL3 gene seem to be recurrent genetic changes [15–18]. At the molecular genetic level, the translocation maps to the TGFBR3 gene at 1p22 and near the MGEAS locus at 10q24. The same translocation also occurs in hemosiderotic fibrolipomatous tumor [15, 16].

Natural history of the tumor is variable, with a high rate of local, often multiple, recurrences [6, 7]. Local recurrence of MIFS is quite high, with rarely distant metastasis [6, 7]. Therefore, close clinical follow-up of patients is needed. In our case, the lesion may initially be considered to be inflammatory and reactive mass because of the location and the presence of many inflammatory cells. In order to avoid local recurrence and repeated surgeries, we should keep in mind slowly growing malignancies when mass locates close to the synovial regions. Regarding the treatment of MIFS, there are no formal standard treatment protocols. Complete excision should be performed whenever this could be achieved in function-preservation. As reported in this case, good results have recently been obtained by a combination therapy of conservative excision and postoperative radiation [19].

In conclusion, MIFS is a rare intermediate (rarely metastasizing) fibroblastic tumor and may easily be confused with many benign lesions. Although the clinical setting of MIFS is now distinctive, the initial diagnosis can be difficult to make. Therefore, surgeons, radiologists, and pathologists must include MIFS in their differential diagnosis of slow-growing tumors to avoid misdiagnosis leading to inappropriate management and delay in appropriate treatment.
